# Validation of Anti-Adeno Associated Virus Serotype rh10 (AAVrh.10) Total and Neutralizing Antibody Immunogenicity Assays

**DOI:** 10.1007/s11095-023-03625-7

**Published:** 2023-10-25

**Authors:** Elizabeth Butala-Flores, Thien Nguyen, Nithya Selvan, Luke Armstrong, Michelle Miller, Lynn Kamen, Todd Lester, Roman Wernyj, Richie Khanna, Jim McNally, Amanda Hays

**Affiliations:** 1 BioAgilytix Labs, Durham, NC USA; 2 Lexeo Therapeutics, New York, NY USA

**Keywords:** AAV, gene therapy, immunogenicity, neutralizing antibodies, total antibodies

## Abstract

Immunogenicity assessment of Adeno-Associated Virus (AAV) vectors is a critical part of gene therapy drug development. Whether the assays are used for inclusion/exclusion criteria or to monitor the safety and efficacy of the gene therapy, they are critical bioanalytical assessments. While total anti-AAV assays are perceived as easier to develop and implement than neutralizing anti-AAV assays, the gene therapy field is still nascent, and it is not yet clear which of the assays should be implemented at what stage of drug development. Recently AAVrh.10 has gained interest for use in gene therapies targeting cardiac, neurological, and other diseases due to its enhanced transduction efficiency. There is limited information on anti-AAVrh.10 antibodies and their clinical impact; thus, the information presented herein documents the validation of both a total antibody assay (TAb) and a neutralizing antibody (NAb) assay for anti-AAVrh.10 antibodies. In this manuscript, the validation was performed in accordance with the 2019 FDA immunogenicity guidance with additional evaluations to comply with CLIA where applicable. The AAVrh.10 TAb and NAb assays were compared in terms of sensitivity, drug tolerance, and precision, along with a concordance analysis using the same individual serum samples. This comparison gave insight into the utility of each format as a screening assay for inclusion into clinical studies.

**Supplementary Information:**

The online version contains supplementary material available at 10.1007/s11095-023-03625-7.

## Introduction

Gene therapies have become an increasingly attractive therapeutic modality in drug development. Adeno-Associated Virus (AAV) gene therapies are promising drug products, especially in uncommon diseases and rare genetic disorders [1, 2]. They also have potential to be used in more common disease indications as a therapeutic in infectious disease [3]. The approach of *in vivo* gene therapy utilizes viral or non-viral delivery of a therapeutic transgene for the treatment of patients who lack a functional endogenous version of the protein under disease conditions. A common viral delivery method for gene therapies is AAVs. AAV vectors are non-enveloped, replication-deficient viruses that can carry a single stranded DNA sequence of up to 4.8 kilo bases [4]. These AAV viral vectors are of particular interest as they are replication-deficient, allowing them to infect and persist in host cells with limited pathogenicity and low risk for random insertion into the host cell genome, making them generally safe as therapeutic products. AAVs are endemic for most human and non-human primate populations and consist of different serotypes that have variable tissue tropisms. Of these serotypes, AAV2, AAV5 and AAV9 are common, well-studied serotypes that are being utilized in many clinical trials for various indications. Outside of naturally occurring AAV serotypes, there have been efforts to modify or engineer recombinant AAVs (rAAVs) to enhance the tropism for specific tissues relevant to different disease indications [5, 6, 7] or to decrease any potential immune responses to these vectors.

Different serotypes are specifically chosen for their preference in targeting certain disease tissue types. For example, in neurological disorders, it is preferable to choose an AAV serotype that can penetrate the blood brain barrier (BBB) to reach the central and peripheral nervous system to deliver a transgene. After intravenous (IV) injection in mice, AAV9 was shown to pass the BBB and transduce various tissues of the central nervous system, including dorsal root ganglia and motor neurons of the spinal cord [8]. Given its capacity to cross the BBB, AAV9 was subsequently used as a gene therapy product for neurological disorders, including the FDA approved AAV gene therapy, Zolgensma®, for spinal muscular atrophy [9]. AAVrh.10 is a serotype that has more recently gained popularity for potential use as a vector in gene therapy delivery for neurological indications. Compared to other AAV serotypes (AAV1, AAV5, and AAV9), AAVrh.10 was shown to have higher transduction and expression in neurons and glial cells in rats [10]. AAVrh.10 showed higher transduction in cells of the spinal cord of mice as compared to other AAV serotypes such as AAV9 [11] and AAV5 and AAV6 [12]. AAVrh.10 also has demonstrated central nervous system (CNS) distribution in non-human primates (NHPs) [13]. Collectively these studies show the potential for AAVrh.10 delivery of gene therapies for treatment of chronic spinal cord injuries and other neurological disorders [14]. With the potential success observed in preclinical studies, AAVrh.10 has also shown promise in clinical studies for gene therapy via intracranial delivery of the therapeutic to treat lysosomal storage diseases [15]. In addition, a study was conducted to demonstrate the biodistribution of AAVrh.10 in NHPs after intravenous infusion using non-invasive imaging indicating distribution of the vector to the liver, heart, and vertebrae [16].

Despite the effectiveness of AAVs in gene therapy, they pose some challenges as delivery vectors due to the natural exposure of various populations to AAVs and the potential for pre-existing immune responses [17, 18, 19]. This is especially a concern for individuals who have high neutralizing antibody titers to AAV serotypes which could lead to reduction in the efficacy of AAV gene therapy by inhibiting the transduction and subsequent impact on protein expression. In addition to the potential impact on efficacy, pre-existing antibodies to AAVs could also have safety implications and unwanted adverse events, such as thrombotic microangiopathy, when subjecting patients to high titer doses of the AAV drug product [20]. Due to these concerns with AAV gene therapies, it is common practice for patients to be screened for inclusion or exclusion from clinical trials for AAV gene therapies based on their anti-AAV titer values or positivity. Assay formats for detection of anti-AAV antibodies include TAb assays or NAb assays. TAb assays measure total anti-AAV antibodies through bridging or sequential immunoassay formats. In contrast, NAb assays are typically cell-based reporter assays that are designed to detect only the anti-AAV antibodies which can inhibit transduction of AAVs and transgene expression. Although considered a more direct measure for efficacy, cell based NAb assays are typically more complex and difficult to implement, and susceptible to inhibition or neutralization from non-antibody factors [26, 36, 37], compared to TAb assays.

In the context of how this translates to clinical studies, it is important to understand the performance of these assays in relation to each other and how they can be used in support of gene therapy studies. An important point to consider when comparing TAb and NAb data for anti-AAV antibodies across different studies is that methods from lab to lab are not harmonized. Assay formats differ between labs and more importantly, the titer cutoff or cut point criteria. Traditional anti-drug antibody assay validations use statistically derived cut points that are based on confidence intervals from validation data to allow for a certain targeted false positive rate [21], whereas assay cutoffs are typically estimations of a threshold criteria that is applied to the assay based on historical data or other criteria. Assay cutoffs are typically set as a value at which screened patients are considered either negative or positive in relation to the specified cutoff value which serves as a threshold for exclusion or inclusion of patients from a clinical trial. These cutoff values used for inclusion and exclusion in clinical studies can vary, with some studies using arbitrary cutoff values and others using titer determined cut points from validation data. For example, for Zolgensma®, the FDA-approved AAV9 gene therapy for treatment of spinal muscular atrophy, an anti-AAV9 total antibody enzyme-linked immunosorbent assay (ELISA) was used for patient enrollment with a cutoff of <1:50 titer [22]. To minimize differences in enrollment and data variability, in clinical studies during drug development, the anti-AAV9 ADA assays were identical and only deployed in one or two laboratories. However, in current clinical usage, the assays used to allow prescribing of Zolgensma® to patients are different from the assays used in the clinical study enrollment during drug development and these assays use a different cutoff. More specifically, one of the labs used for patients treated in clinical practice in the United States defines patients with titers of <1:25 as being seronegative, whereas the labs used in the clinical trial studies defined patients with titers of <1:50 as seronegative. The data suggests different assay cutoffs, a result of the differences in assay cut point determinations, inhibit comparability of data from individuals between assays [22]. The lack of harmonization in assay usage and variability in clinical cutoffs is also seen across clinical studies for different indications, where cutoffs range from <1:1 to <1:400 for NAb or TAb assays [23]. With the variability in data amongst clinical studies, it remains unknown what a suitable clinical cutoff would be for an anti-AAV assay and may be specific for each individual drug product.

In this manuscript, we present validation of a TAb assay and a NAb assay for the detection of anti-AAVrh.10 antibodies in human serum. The assays are compared, including a concordance of the results from both assays of the same set of samples, and the results are used to make a case for how these assays could be used for inclusion and exclusion into AAVrh.10 gene therapy clinical studies.

## Materials and Methods

### Serum Samples

All normal human serum samples were collected from whole blood. All individual serum samples were purchased from BioIVT (Hicksville, NY, USA) and stored at -20°C or colder until use. Serum samples for the NAb assay were heat inactivated in a 56°C water bath prior to use. Serum from female individual donors were not included in method validation due to a supply chain shortage of serum during the COVID-19 pandemic.

### Negative Controls

A pooled serum sample (NC, Negative Control) for each assay was prepared by combining equal volumes of selected normal individuals predicted to be negative for anti-AAVrh.10 antibodies in the described assay. The samples chosen were predicted to be negative since a cut point was not yet determined in method development. The NC pools were tested prior to assay validation to demonstrate minimal reactivity (data not shown).

### Positive Controls

For the TAb assay, PC (positive control) samples were prepared by diluting the surrogate positive control (SPC) anti-AAV8 mouse monoclonal antibody that cross reacts with anti-AAVrh.10 (Progen, clone ADK8) in the NC serum pool. HPC (high positive control) was prepared at 500 ng/mL in neat matrix, MPC (mid positive control) was prepared at 250 ng/mL, and LPC1 (low positive control 1) and LPC2 (low positive control 2) were prepared at 100 and 35 ng/mL, respectively. The LPC concentration was set based on a 1% false positive rate (FPR) in assay qualification.

Due to a steep dose-response curve observed in method development, it was challenging to make selections of positive control concentrations with the SPC. Therefore, for the NAb assay, PC samples were prepared with dilutions of positive AAVrh.10 serum rather than the SPC and prepared by diluting a positive anti-AAVrh.10 serum sample in negative pooled serum. HPC was prepared at a 50-fold dilution, MPC was prepared at 200-fold dilution, and LPC1 and LPC2 were prepared at 300-fold and 400-fold dilutions, respectively. The controls were stored in single use aliquots. To ensure that the LPC dilutions were suitable for the NAb assay, a one-sided upper limit prediction interval was calculated for the LPC1 and LPC2. The SPC (clone ADK8) was also used for additional characterization of the NAb assay. For both assays, the normalized signal was determined by dividing the raw signal from each well by the mean of the NC on each assay plate. For the TAb assay the normalized signal is represented by signal to noise (S/N). For the NAb assay the normalized signal is represented by normalized luminescence (normLum) units.

### TAb Assay

MSD GOLD Sulfo-Tag NHS-Ester was used for labeling AAVrh.10 with ruthenium and manufacturer instructions were followed for the labeling procedure. Briefly AAVrh.10-Luc was acquired from UMass Gene Therapy Center and Vector Core. Vector underwent Zeba column buffer exchange into conjugation buffer (1X PBS pH 7.9) and quantitated using Progen AAVrh.10 Titration ELISA (Progen, Cat# PRAAV10). Ruthenium labeling occurred at 16 nmol label per 2 x 10^12^ AAVrh.10 capsid. Ruthenylated AAVrh.10 was isolated by Zeba column buffer exchange into storage buffer (1X PBS pH 7.4, 0.05% sodium azide). Finally, labeled vector was quantitated by Progen AAVrh.10 Titration ELISA prior to use in TAb assays.

For a sequential bridging electro-chemiluminescent assay, unlabeled AAVrh.10-Luc diluted in PBS was immobilized to a Meso Scale Discovery multiarray standard 96-well plate (MSD, USA). The plates were sealed with microplate sealers and incubated in a 4°C refrigerator for 12–20h to allow for passive adsorption of AAVrh.10 to the surface of the plates. The next day, the plates were washed three times using 300 µL of PBS-T (phosphate buffered saline/0.05% Tween 20) buffer with an automated plate washer (Biotek, ELx405). The plate was then blocked with 3% MSD Blocker A in PBS-T (MSD, USA) for 1 hour with shaking at 600 rpm to block non-specific signal from detection. Human serum samples and controls were diluted to a minimum required dilution (MRD) of 1:75 in 1% Blocker A in PBS-T and incubated in the blocked MSD plate for 1.5 hours with shaking at 600 rpm. Ruthenium labeled AAVrh.10-Luc was added to the plate and incubated for 1 hour while shaking at 600 rpm. The plate was washed three times with PBS-T with an automated plate washer, then 2X MSD Read Buffer T was added to the plate and read on the MSD S600 Sector Imager. A signal within the well correlates with the presence of TAb in the sample. A sample with S/N greater than or equal to the screening cut point (SCP) is considered presumptive positive. A sample with S/N less than the SCP is considered presumptive negative.

### NAb Assay

For the cell-based luminescent neutralizing antibody assay, an AAVrh.10 vector containing firefly luciferase reporter gene, was applied to a transduction permissive cell line, 2V6.11. 2V6.11 cells were derived from HEK293 (human embryonic kidney cell line) cells and were stably transfected with adenoviral genes under control of an ecdysone-responsive promoter. In the assay, 2V6.11 cells were cultured in Dulbecco’s Modified Eagle Medium supplemented with 10% FBS and penicillin-streptomycin in 75 or 150 cm^2^ flasks. On the day of the assay, the cells were washed with DPBS and detached with TrypLE reagent (Gibco, USA). The cells were diluted in supplemented phenol free DMEM, counted, and centrifuged at 125 x g for 5 minutes. The cell pellet was resuspended in media supplemented with 1 μg/mL Ponasterone A (Invitrogen, USA) to a density of 6x10^5^ cells/mL. The cell suspension was dispensed into a white 96-well Poly-D-Lysine coated plate, such that each well contained 30,000 cells. The plate was placed in a humidity chamber and incubated overnight for 18-26 hours in a 37°C/5% CO_2_ incubator. The next day, human serum samples and assay controls were diluted 2-fold in supplemented phenol free DMEM. The AAVrh.10-Luc vector was diluted in media supplemented with Ponasterone A and mixed in equal volume with the human serum samples and assay controls for a final assay MRD of 1:4. The co-incubation plate(s) were set on a plate shaker at 300-400 RPM for 60-75 minutes at room temperature. Media was aspirated from the cell plate and the vector/serum mixture was added to the cell plate to achieve a final multiplicity of infection (MOI) of 5,000 vector genomes (vg) per cell seeded. The cell plate was transferred to a 37°C, 5% CO_2_ incubator. After 24 hours of incubation, the cell plate was moved to ambient temperature in the dark, One-Glo reagent (Promega, USA) was added and the plate was incubated for a minimum of 5 minutes before reading luminescence on a Biotek Cytation 3. The presence of NAb in human serum samples inhibits the luminescence-generating activity of the virus, thereby reducing signal in the assay. The absence or presence of NAb was determined by comparing the signal in the assay to a statistically derived assay cut point. A sample with normalized signal less than or equal to the SCP is considered presumptive positive. A sample with normalized signal greater than the SCP is considered presumptive negative.

### Statistical Analysis

All graphs were generated using GraphPad Prism (version 3.02) or JMP Statistical Software (version 12). Cut points were determined using JMP SAS Statistical Software Version 12. Outliers were assessed using the linear mixed effects model; analytical outliers were identified with Tukey Box Plot outlier criteria from the model’s conditional residuals and biological outliers were identified with Best Linear Unbiased Predictor (BLUP). The Cohen’s Kappa test was used to determine statistical concordance between TAb and NAb assays.

## Results

### Minimum Required Dilution (MRD) and Vector Titration Curve

**Fig. 1:**
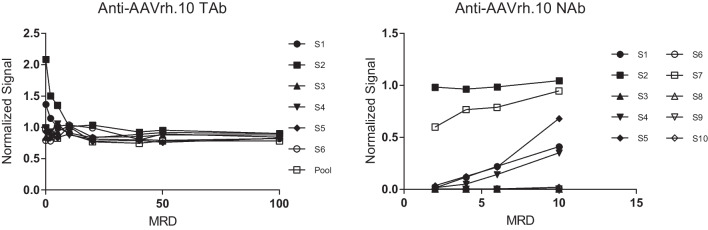
Minimum Required Dilution (MRD). In method development of the anti-AAVrh.10 TAb assay, six normal human serum samples (S1-S6) and a serum pool were assayed neat and at serum dilutions of 2, 5, 10, 20, 40, 50, and 100. In method development of the anti-AAVrh.10 NAb assay, ten normal human serum samples (S1-S10) were assayed at a 2-, 4-, 6-, and 10-fold dilution. An MRD of 1:75 was chosen for the TAb assay, and an MRD of 1:4 was chosen for the NAb assay that showed appropriate removal of matrix interference while achieving sufficient assay sensitivity.

To determine the optimal MRD for the TAb assay, six individual serum samples (designated S1-S6) and a pooled serum sample were tested in the assay at multiple dilutions. In subsequent experiments, sensitivity and MRD were also assessed using titration of the SPC at multiple MRDs. The normalized signal of the individual six serum samples along with the pooled serum sample was evaluated across all dilutions. The normalized signal varied among the samples at lower MRD dilutions indicating variability in response that suggests matrix interference. The normalized signal appears to level out across all serum samples tested between the 1:50 and 1:100 dilution demonstrating a consensus in signal once the interference effects were sufficiently diluted out. The results suggested a suitable MRD of 1:75 for the assay (Fig. 1).

For the NAb assay, the MRD was determined by diluting ten normal serum samples (designated Sample 1 to Sample 10 or S1 to S10) at 1:2, 1:4, 1:6, and 1:10 dilutions (Fig. 1). The samples used for MRD assessment in the NAb assay were different donor serum samples that were used in the TAb assay MRD assessment experiment. Most of the serum samples that were tested had a consistent signal when normalized to assay buffer across all dilutions. Sample 2 was the only sample with a normalized signal close to 1.00 across all MRD dilutions. Sample 7 showed a trend of increasing normalized signal approaching 1.00 with increasing MRD, and sample 5 appeared to have a low normalized signal indicating presence of NAbs and a decrease in signal by MRD 10. Taking these data into account, early indications of matrix interference were not observed and an MRD of 1:4 was chosen for the assay.

**Fig. 2:**
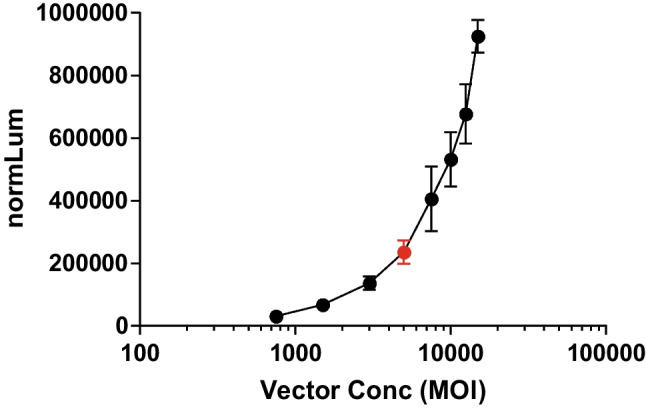
Vector titration curve for the anti-AAVrh.10 neutralizing antibody assay. The vector titration response curve shown represents the mean response from three dose response curves evaluated by two analysts on three different days. The vector titration curves were prepared by diluting AAVrh.10-Luc working stock to give eight concentrations ranging from 750 to 15000 MOI (vg/cell). The results indicate that the selected AAVrh.10 Luc vector concentration at 5000 multiplicity of infection (MOI), depicted in the red circle, provides an acceptable assay signal window at the MRD 1:4.

The vector titration response curve was evaluated by generating a total of three dose response curves in the level of matrix equal to the MRD (1:4) prepared by two analysts on three different days. The vector titration curves were prepared by diluting AAVrh.10-Luc working stock to give eight concentrations ranging from 750 to 15000 MOI (multiplicity of infection) (vg/cell). Vector titration curve results are shown in Fig. 2. The assay vector concentration resulted in a mean luminescence signal range of 193,647 to 266,517. The results indicate that the selected AAVrh.10 concentration (5000 MOI) provides an acceptable assay signal window.

### Assay Cut Points

Screen and titer assays were developed and validated for both the TAb and NAb assays. Given the prevalence of pre-existing immunity to AAV as a result of previous exposure, and high percent positive rates within subjects is expected, it eliminates the necessity of a confirmatory cut point. Furthermore, due to the high pre-existing positive signal, the amount of drug (AAV capsid) required to produce a reasonable % inhibition window in a confirmatory assay is likely not sustainable from a resource perspective. It usually requires capsid at concentrations 10-100 times the plate coating concentrations which translates to 10^13^ vg/mL quantities. Overall, analysis of samples using a screen and titer methodology only provides sufficient evidence of a robust positive response and a specific response which will titrate out according to expected dilutions. Therefore, a confirmatory component was not evaluated in these assays.

A preliminary screen for TAb-negative samples was made prior to validation by testing a panel of 140 individual serum samples at an MRD of 10. For the TAb assay, a screening cut point (SCP) and titer cut point (TCP), were established by testing 56 individual human serum samples at MRD 75 that were assayed seven times in duplicate, by two analysts over four days in a balanced design setup. Each plate for cut point determination included six replicates (three sets) of the NC and four replicates (two sets) of a high and low control. The mean raw signal of the NC was used to normalize responses of the individual serum samples. The cut point was determined as described in [24]. The distribution of conditional residual values obtained from the model was utilized to identify analytical outliers using the Tukey box-plot outlier criteria. A total of 17 analytical outliers were identified and removed. The distribution of individual subject best linear unbiased predictor (BLUP) values was next evaluated to identify samples as biological outliers by the same criteria used for the conditional residuals. Six samples were identified as biological outliers. Following outlier removal, the distribution of the remaining log-10 transformed normalized dataset (n=327) was evaluated using Shapiro-Wilk to test for normality of the distribution (Table I). The tests for normality passed (Shapiro-Wilk p-value = 0.1160) indicating that the distribution was normal, and a 5% false positive rate (FPR) was targeted to yield a SCP of 1.21. The TCP was determined with a target for a 0.1% FPR and was determined to be 1.40.

**Table I:** Assay Cut Points

TAb Cut Point Parameters		Value		NAb Cut Point Parameters		Value	
Total Number of Assay Values (56 samples × 7 runs)		392		Total Number of Assay Values (46 samples × 6 runs)		276	
Values excluded for imprecision (%CV >20%)		8		Values excluded for imprecision (%CV >25%)		3	
Analytical Outliers		17		Analytical Outliers		12	
Biological Outliers		40		Biological Outliers		23	
Total Values Used in Screening Cut Point Analysis		327		Total Values Used in Screening Cut Point Analysis		238	
TAb Cut Point Determination							
Y	Quantile	Cut Point Estimate	Lower 90%	Upper 90%	Actual Coverage	Log10	Cut Point Estimate
S/N (Screening)	5%	0.08636	0.07555	0.09691	90.22	0.0820	1.21
S/N (Titer)	0.1%	0.16905	0.1427	0.17208	-	0.1453	1.40
NAb Cut Point Determination							
Y	Quantile	Cut Point Estimate	Lower 95%	Upper 95%	Actual Coverage	Continuous Fit	Quantile Cut Point Estimate
Mean NormLum	1%	0.587	0.552	1.931	90.855	Not Normal	0.587
Mean NormLum	5%	0.678	0.637	0.717	96.172	Not Normal	0.678

Samples were assayed prior to validation in a preliminary screening in order to identify NAb-negative samples to be used for cut point determination in validation. In the preliminary screening 140 heat inactivated samples were tested at a dilution of 2 to 4-fold. In validation, the screening assay cut point was established by testing a selection of 46 human serum samples predicted to be NAb-negative from the preliminary screen (samples with normLum values greater than 0.6) six times in duplicate, by two analysts over 4 days. A minimum of 6 replicates (3 sets of technical duplicates) of the NC were included on each plate. The mean value of the NC replicates was used to normalize the cut point sample responses. Data was normalized to the NC by dividing the luminescence signal of each sample result by the mean NC luminescence signal on each individual assay plate to generate the normLum for each sample or control. Additionally, each cut point plate contained 6 replicates (3 sets of technical duplicates) of the HPC, MPC, LPC1, and LPC2. Analytical and biological outliers from this dataset were identified using a linear-mixed-effects model approach. The distribution of conditional residual values from the model were evaluated to identify ‘analytical’ statistical outliers as values above the 75th percentile plus 1.5 times the inter-quartile range or below the 25th percentile minus 1.5 times the inter-quartile range. A total of 12 analytical outliers were collectively identified and removed following iterative Tukey outlier boxplot criteria. From the remaining data, the distribution of individual subject best linear unbiased predictor (BLUP) values was examined to identify subjects as ‘biological’ statistical outliers by applying the same criterion used for conditional residuals. In total, 38 values were removed as technical, analytical, and biological outliers. The remaining 238 values were used for determining the cut point. After outlier exclusion, the distribution of the remaining, untransformed normalized results was examined, and normality assessed using Shapiro-Wilks test.

Since the average run specific cut points did not yield a suitable cut point, quantile estimates (non-parametric) at 1% and 5% FPR were calculated with a 95% confidence interval for the entire data set. The 1^st^ percentile point estimate (PE) gave a CP estimate of 0.587, and the 5^th^ percentile PE gave a CP estimate of 0.678 with acceptable levels of actual coverage for both. Applying the 1^st^ percentile PE CP of 0.587 to the 238 included determinations resulted in two determinations falling below the CP point for a FPR of 0.8%. Applying the 5^th^ percentile PE CP estimate of 0.678 to the 238 included determinations resulted in 12 determinations falling below the cut point for a FPR of 5.0%. Two cut point estimates (0.587 and 0.678) were used to evaluate the results of the sensitivity experiment and based on the results; the 1% percentile point estimate CP (0.587) was selected for continued use in the assay (Table I). Since the 1% FPR was used to determine the screening cut point, the same cut point (0.587) was also used as the titer cut point in the NAb assay.

### Sensitivity and Drug Tolerance

**Fig. 3:**
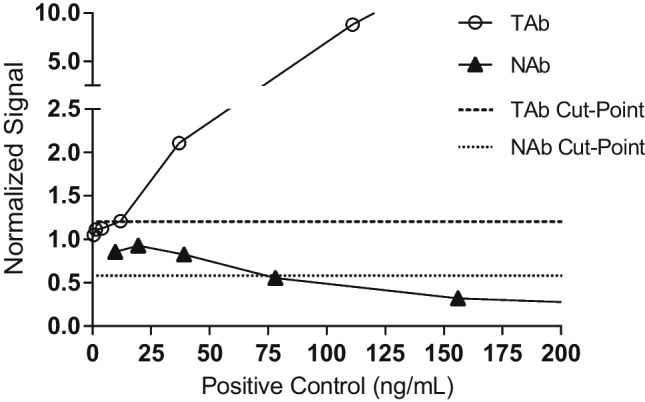
TAb and NAb cut point estimation. Sensitivity was evaluated in the anti-AAVrh.10 TAb assay by assaying a series of dilutions (open circles) of the positive control at the 1:75 MRD. The data shown are the mean of six independent runs. The TAb cut-point is depicted by the dashed line (1.21). Sensitivity of the anti-AAVrh.10 NAb assay was determined by assaying the PC at a series of dilutions (closed triangles) at the 1:4 MRD. The data shown are the mean of six independent runs. The NAb cut-point is depicted by the dotted line (0.587)

Sensitivity for both the total and neutralizing antibody assays was determined based on titrations of the surrogate positive control (clone ADK8). The TAb assay sensitivity was calculated from 6 independent runs with the SPC at eight concentrations ranging from 1000 ng/mL to 0.5 ng/mL diluted to the MRD in assay buffer. Sensitivity was defined as the interpolated concentration value, x, at which the 4PL curve fit of the PC titration cross the assay cut point in each run. Values from the individual titrations are shown in Table II. The pooled curves are shown in Fig. 3. The sensitivity of the screening TAb assay was calculated from the mean of the interpolated concentration of the six titration curves, and was determined to be 8.9 ng/mL, meeting the target acceptance criteria of sensitivity less than or equal to 100 ng/mL [38]. Estimated concentrations of the LPC assay control were calculated from these runs and yielded a concentration of 11.5 ng/mL (Table II). Concentrations of 16.5 ng/mL cut point LPC (cpLPC) were tested later in the validation to establish an LPC concentration with a 1% failure rate in the assay. However, based upon inter-assay precision, cpLPC frequently had S/N values lower than the SCP with a failure rate of 80%. Therefore, an LPC with a concentration of 35 ng/mL which was consistently higher than the SCP was chosen as the LPC concentration for sample analysis.

**Table II:** TAb Sensitivity

SPC (ng/mL)	Run 1		Run 2		Run 3		Run 4		Run 5		Run 6	
	S/N	%CV	S/N	%CV	S/N	%CV	S/N	%CV	S/N	%CV	S/N	%CV
1000	**60.63**	1.9	**106.14**	4.3	**88.10**	8.4	**86.20**	2.3	**76.89**	3.5	**107.41**	0.9
333	**25.78**	0.8	**59.92**	3.2	**27.74**	13.6	**44.49**	3.6	**34.90**	24.4	**30.90**	1.3
111	**5.77**	2.0	**15.13**	6.1	**5.81**	1.4	**10.26**	2.6	**9.33**	0.8	**6.53**	1.9
37	**1.74**	4.8	**3.26**	2.6	**1.55**	15.0	**2.19**	0.4	**2.05**	5.4	**1.86**	1.6
12	**1.31**	21.0	**1.40**	13.6	1.07	9.9	1.13	13.4	1.15	1.0	1.19	3.1
4.1	**1.39**	4.2	**1.24**	6.4	1.06	6.3	1.00	11.3	1.01	1.2	1.06	1.4
1.4	**1.26**	22.3	1.08	5.7	1.13	2.9	1.00	27.3	1.01	2.9	1.20	1.2
0.5	0.99	3.0	1.11	2.2	1.24	25.5	0.91	10.8	1.00	5.8	-	-

Bold represents values > Screening Cut Point of 1.21

The NAb assay sensitivity was calculated from 6 individual runs with the SPC stock at 8 concentrations ranging from 1250 ng/mL to 9.77 ng/mL. Sensitivity was defined as the interpolated concentration value x, at which the 4PL curve fit of the antibody titration crossed the assay cut point (based on 1% and 5% FPR) in each run. Values from the individual titrations are shown in Table III. The pooled curves are shown in Fig. 3. The sensitivity of the NAb assay was calculated from the mean of the interpolated concentration of the six titration curves. For the titrations set at the 1% CP (0.587), the mean sensitivity was 77.7 ng/mL. For the titrations set at the 5% CP (0.678) the mean sensitivity was 61.2 ng/mL. Both values met the target acceptance criteria of sensitivity less than or equal to 500 ng/mL [38].

**Table III:** NAb Sensitivity

NAb Sensitivity												
SPC (ng/mL)	Run 1		Run 2		Run 3		Run 4		Run 5		Run 6	
	Mean normLum	%CV	Mean normLum	%CV	Mean normLum	%CV	Mean normLum	%CV	Mean normLum	%CV	Mean normLum	%CV
1250	**0.083**	11.7	**0.107**	7.9	**0.073**	0.0	**0.077**	17.6	**0.054**	19.1	**0.084**	1.0
625	**0.141**	7.3	**0.153**	2.1	**0.111**	6.7	**0.118**	4.9	**0.090**	13.7	**0.088**	16.0
313	**0.217**	4.7	**0.234**	17.6	**0.177**	8.1	**0.149**	20.4	**0.146**	12.6	**0.129**	12.8
156	**0.409**	7.9	**0.412**	4.4	**0.345**	4.0	**0.241**	7.7	**0.309**	11.3	**0.208**	14.0
78.1	0.691	2.3	0.674	0.4	**0.534**	2.6	**0.485**	0.9	**0.575**	0.7	**0.362**	1.4
39.1	0.850	8.3	0.908	1.7	0.813	1.6	0.747	7.3	0.964	2.2	0.663	3.5
19.5	0.998	5.2	0.964	8.6	0.933	0.2	0.846	5.6	0.970	2.9	0.846	11.8
9.77	0.954	1.0	0.558	21.0	0.964	8.0	0.921	5.9	0.987	5.4	0.752	11.5

Bold represents values > Screening Cut Point of 0.587

While sensitivity was determined using the SPC, assay controls for the NAb assay were prepared with dilutions of positive AAVrh.10 serum rather than the SPC. During method development, the dose-response curve for the SPC was very steep, proving a challenge to make selections of PC concentrations. It was decided to use positive serum samples as the preferable option for a relevant control, especially since the SPC is intended to be a surrogate and is a monoclonal antibody which does not reflect the antibodies that would be present in patient samples. Two dilutions of this serum sample, LPC1 (300-fold dilution) and LPC2 (400-fold dilution), were determined from titrations run during assay development. To confirm that these LPC dilutions were suitable for the assay, a one-sided upper limit prediction interval (PI,0.99 1-Alpha value) was calculated for the LPC1 and LPC2 using a total of 18 mean normalized signal values (data not shown). Due to the LPC1 0.99 PI value being too low as compared to the FPR cut point of 1% (0.587), the LPC2 control, which had a 0.99 PI value of 0.569, which was closer to the 1% FPR cut point, and was selected as the LPC assay control for sample analysis.

Drug tolerance for the TAb assay was evaluated by testing the HPC (500 ng/mL), MPC (250 ng/mL), LPC1 (100 ng/mL), and LPC2 (35 ng/mL) spiked with seven concentrations of AAVrh.10-empty (1.80 E+13, 3.60 E+12, 7.20 E+11, 1.44 E+11, 2.88 E+10, 5.76 E+09, 1.15 E+09, and 0 vp/mL) run in duplicate. The level of drug tolerance was defined as the ability of the TAb assay to detect the SPC as positive relative to the SCP. Results including the concentrations of drug and SPC are shown in Table IV. The HPC, MPC, and LPC1 were tolerant up to 7.20E+11 vp/mL of drug. LPC2 was tolerant up to 1.44E+11 viral particles (vp)/mL of the drug.

**Table IV:** TAb Drug Tolerance

AAVrh.10 (vp/mL)	AAVrh.10 (vp/well)	500 ng/mL HPC		250 ng/mL MPC		100 ng/mL LPC1		35 ng/mL LPC2	
		S/N	% CV	S/N	% CV	S/N	% CV	S/N	% CV
1.80 E+13	1.20 E+10	1.02	6.3	0.80	3.8	0.77	6.1	0.77	6.7
3.60 E+12	2.40 E+09	0.88	19.5	0.82	4.7	0.76	1.7	0.73	4.1
7.20 E+11	4.80 E+08	0.90	0.0	0.86	12.6	0.75	8.6	0.74	3.5
1.44 E+11	9.60 E+07	20.40	12.4	8.13	0.4	1.44	5.4	0.90	8.1
2.88 E+10	1.92 E+07	46.44	0.9	22.15	0.5	6.29	7.9	1.28	12.1
5.76 E+09	3.84 E+06	51.09	8.7	28.39	1.7	8.03	3.3	1.91	1.6
1.15 E+09	7.68 E+05	52.27	5.4	28.67	1.2	7.75	5.2	1.90	17.2
0	0	51.28	4.2	26.66	0.4	8.52	9.0	1.85	10.5

Drug tolerance for the NAb assay was evaluated by testing five concentrations of the SPC (1100, 250, 150, 100, and 0 ng/mL) spiked with six concentrations of empty AAVrh.10 Vector (0, 3333, 5005, 7508, 10014, and 15015 MOI). Drug tolerance samples were tested in duplicate in one run (over two plates) by one analyst. Data for the drug tolerance samples are shown in Table V. As indicated by the ability to detect the SPC as positive relative to the CP, SPC levels at 1100, 250, 150 ng/mL were tolerant up to 15015 MOI of AAVrh.10-empty, and the 100 ng/mL SPC level was tolerant up to 10014 MOI of AAVrh.10. Specificity was demonstrated by an increase in assay signal in all four SPC concentrations spiked with AAVrh.10-empty and an increase in signal for the NC spiked with AAVrh10-empty relative same controls tested without AAVrh.10-empty. Although drug tolerance was evaluated in both assay validations, it would likely not impact antibody detection or titration in the assays given the virus is typically shed within weeks as compared to other biotherapeutics with longer half lives in circulation [25].

**Table V:** NAb Drug Tolerance

AAVrh.10 (vg/uL)	AAVrh.10 MOI (vg/cell)	1100 ng/mL SPC		250 ng/mL SPC		150 ng/mL SPC		100 ng/mL SPC		0 ng/mL	
		Mean normLum	%CV	Mean normLum	%CV	Mean normLum	%CV	Mean normLum	%CV	Mean normLum	%CV
4.50E+06	15015	0.093	7.3	0.298	13.7	0.487	17.4	0.678	9.1	1.139	5.5
3.00E+06	10014	0.088	20.1	0.202	18.5	0.369	4.7	0.527	8.2	1.043	5.3
2.25E+06	7508	0.088	2.6	0.153	1.0	0.269	7.1	0.407	5.0	1.007	0.3
1.50E+06	5005	0.081	8.8	0.129	3.8	0.279	18.2	0.353	7.6	1.060	3.0
7.51E+05	3333	0.079	37.1	0.117	12.0	0.230	1.3	0.317	12.1	0.978	5.1
0	0	0.075	22.7	0.140	2.2	0.237	10.8	0.275	1.2	0.833	2.3

### Assay Precision

Inter- and intra-assay precision was assessed based on the performance of the HPC, MPC, LPC controls prepared in serum in the screening assay for both TAb and NAb assays. Inter-assay precision for both TAb and NAb assays was evaluated using the mean normalized signals of the HPC, MPC, LPC1, LPC2 and NC from all acceptable validation runs. For the NC, the mean normalized value from each plate was defined as 1.00; therefore, inter-assay precision was generated from the raw signal values of all individuals replicates on all plates.

Inter-assay precision of all PCs across all plates by all analysts met target acceptance criteria in the TAb assay. The grand CV values for the PC mean normalized signals across all validation runs were 27.2%, 24.8%, 24.3% and 17.4% for the HPC, MPC, LPC1 and LPC2, respectively. Although the HPC, MPC and LPC1 had inter-assay CV greater than 20%, they screened positive relative to the assay cut point and fell within rank order. The grand CV value for the cpLPC was 11.4%. Inter-assay precision of the NC met target acceptance criteria for the raw signal values, with an overall CV value of 9.8%. The NAb assay inter-assay precision of all PCs across all plates by all analysts met target acceptance criteria. The grand CV values for the PC mean normLum signals across 18 plates in 9 runs were 13.2%, 18.7%, 16.8% and 11.6% for the HPC, MPC, LPC1 and LPC2, respectively. Inter-assay precision of the NC met target acceptance criteria for the normalized signal values, with a CV value of 10.9%.

Intra-assay precision for both TAb and NAb assays were evaluated in one intra-assay run including six independent replicates of the HPC, MPC, LPC1, LPC2 and NC. The results for intra-assay precision of the TAb assay met the target acceptance criteria with CV values < 20% for the HPC, MPC, LPC1, LPC2 and NC, with the exception from one out of six runs where HPC replicates had intra-assay CV of 64.6%. Regardless, the mean normalized single of the HPC, MPC, LPC1 and LPC2 screened positive and fell within rank order. The results for intra-assay precision of the NAb assay met the target acceptance criteria with CV values of 13.8%, 8.7%, 6.7%, 4.6%, and 8.1% for the HPC, MPC, LPC1, LPC2 and NC, respectively. The controls in the total and neutralizing antibody assays demonstrated robust inter- and intra-assay precision. Precision data for both assays are summarized in Table VI.

**Table VI:** Assay Precision

	TAb Intra-Assay								TAb Inter-Assay	
Control (ng/mL)	Rep 1	Rep 2	Rep 3	Rep 4	Rep 5	Rep 6	S/N	CV (%)	S/N	CV (%)
HPC (500)	58.95	54.82	55.72	54.52	59.01	53.16	55.95	4.1	61.00	27.2
MPC (25)	31.23	29.13	30.91	28.91	32.76	31.46	30.73	4.8	33.93	24.8
LPC1 (100)	10.06	8.14	8.86	8.82	9.15	8.22	8.88	7.9	9.45	24.3
LPC2 (35)	2.34	2.28	2.41	2.32	2.22	2.30	2.31	2.7	2.35	17.4
cpLPC (16.5)	-	-	-	-	-	-	-	-	1.07	11.4
NC							1.00	18.2	1.00	9.8
	NAb Intra-Assay								NAb Inter-Assay	
Control	Rep 1	Rep 2	Rep 3	Rep 4	Rep 5	Rep 6	Mean normLum	CV (%)	Mean normLum	CV (%)
HPC 50-fold	0.019	0.024	0.024	0.024	0.017	0.023	0.022	13.8	0.024	13.2
MPC 200-fold	0.143	0.166	0.159	0.167	0.173	0.186	0.166	8.7	0.206	18.7
LPC1 300-fold	0.305	0.28	0.28	0.309	0.257	0.287	0.286	6.7	0.345	16.8
LPC2 400-fold	0.381	0.38	0.383	0.421	0.372	0.4	0.389	4.6	0.435	11.6
NC	1.066	0.956	0.939	1.133	0.967	0.938	1.000	8.1	1.000	10.9

*Precision for NC determined from 15 replicates

Titer precision was evaluated in the TAb assay by preparing and diluting the HPC, MPC, and LPC1 and LPC2 controls in 3-fold serial dilutions in normal serum pool to give 7-8 concentrations ranging from 500 to 0.02 ng/mL. The titer value was defined as the last dilution that generated a signal above the titer cut point of 1.40. The titer was reported as the MRD, which was 1:75, multiplied by the last dilution with a mean normalized signal greater than or equal to the screening cut point. Titer values were within one dilution of the most frequently observed titer value. Precision of titer for the neutralizing antibody assay was determined by evaluating 2-fold serial dilutions of the positive serum samples diluted in blank matrix pool until below the respective assay screening cut point. All diluted samples had a titer within one dilution of the nominal titer between two different operators (data not shown).

### Selectivity

**Fig. 4:**
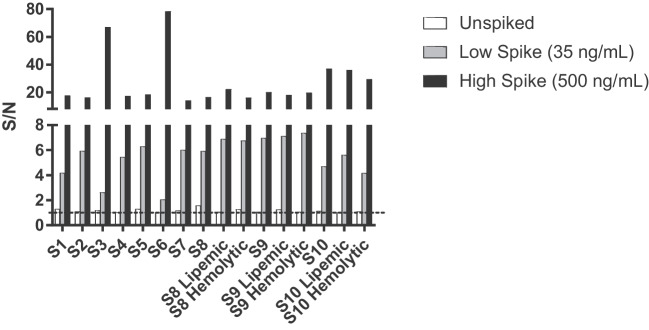
Selectivity assessment in the anti-AAVrh.10 total antibody assay. Ten normal human serum samples (S1-S10) were either left unspiked (white bars), spiked at a low concentration of positive control at 35 ng/mL (grey bars), or spiked at a high concentration of positive control at 500 ng/mL (black bars) and assayed in the screening assay. Samples 8, 9 and 10 were also assayed as lipemic and hemolyzed samples. Hemolyzed samples were prepared by spiking normal serum with 2 mg/mL human hemoglobin and lipemic samples were prepared by spiking normal serum with 4 mg/mL Intralipid. The dashed line represents the screening cut-point at 1.21.

To evaluate matrix interference by assessing selectivity in the TAb assay, ten negative individual serum samples were unspiked, spiked at the HPC (500 ng/mL) or the LPC (35 ng/mL) and evaluated in the screening assay. Ten out of ten (100%) of high- and low-spiked individual samples screened positive in the screening assay and fell in rank order with acceptable CV. Nine (9) out of 10 (90%) unspiked individual samples tested negative in the screening assay with acceptable CV. Selectivity was also evaluated in three hemolyzed and three lipemic human serum samples, unspiked or spiked at the HPC and LPC levels. All hemolyzed and lipemic samples that were spiked at the high or low positive control level, screened positive in the assay. The selectivity data is presented in Fig. 4.

**Fig. 5:**
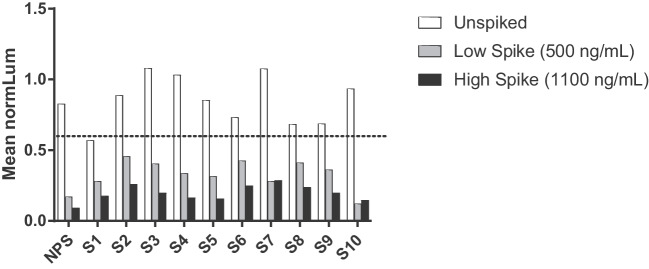
Selectivity assessment in the anti-AAVrh.10 neutralizing antibody assay a pooled serum sample (NPS) and ten normal human serum samples (S1-S10) were either left unspiked (white bars), spiked at a low concentration of positive control at 500 ng/mL (grey bars), or spiked at a high concentration of positive control at 1100 ng/mL (black bars) and assayed in the screening assay. The dashed line represents the screening cut-point at 0.587.

To evaluate matrix selectivity in the NAb assay, 10 negative individual normal human serum samples were spiked with the surrogate positive control at low (500 ng/mL) and high (1100 ng/mL) concentrations. The selectivity samples were also tested unspiked (0 ng/mL SPC) in the same assay. Results for selectivity in human serum samples are presented in Fig. 5. The selectivity assessment passed, with 8 of 10 samples (80%) meeting all target acceptance criteria. All high-spike and all low-spike selectivity samples tested positive (normLum ≤ 0.587 SCP) in the assay; two selectivity samples did not rank order due to high CV values of the low spike sample. Nine (9) out of 10 (90%) of unspiked samples tested negative. Assessment of hemolyzed and lipemic samples were not performed in the NAb validation.

#### Concordance of TAb and NAb Results

**Fig. 6:**
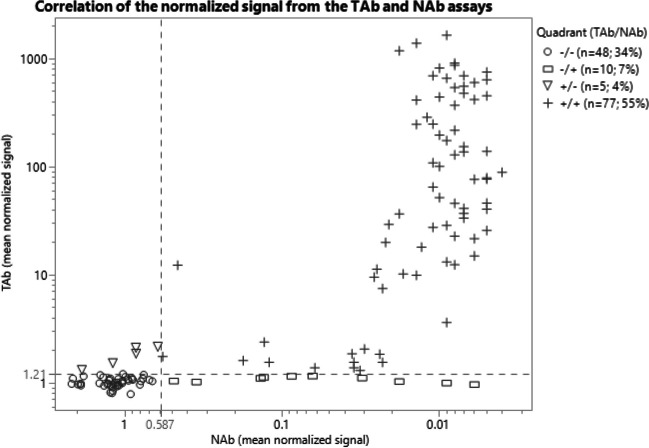
Correlation of anti-AAVrh.10 total antibodies and neutralizing antibodies in human serum. One hundred and forty serum samples were assayed for AAVrh.10 specific antibodies at a serum dilution of 1:75 in the total antibody assay and 1:4 in the cell-based neutralizing antibody assay. Cut point values for each assay are shown by the dashed lines. Samples that screened positive or negative in both or either assay fall into their respective quadrant and are denoted by the symbol corresponding to the figure legend. A Cohen’s Kappa coefficient was determined to be 0.78.

**Fig. 7:**
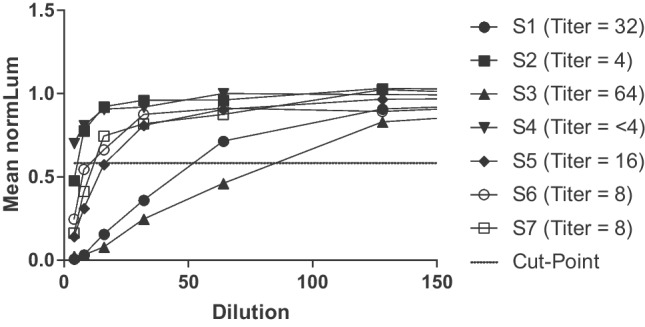
Titration of positive samples in the anti-AAVrh.10 neutralizing antibody assay. Seven of the ten normal serum samples that screened positive in the anti-AAVrh.10 (S1-S7) NAb assay but negative in the TAb assay were titrated in the NAb assay. Each sample was diluted in normal human serum pool in a two-fold dilution series, then diluted 1:2 in assay media before being mixed 1:2 with the AAVrh.10-Luc vector to achieve the assay MRD of 4. Each sample titer curve is displayed with different symbols and shown on the graph. The titer for each sample that crossed the cut-point is displayed next to each sample in the graph legend.

An analysis was performed to understand the agreement or concordance in response between samples that were evaluated in both the TAb and NAb assays (Fig. 6). The normalized responses from 140 normal serum samples were plotted along with the respective TAb validation screening cut point of 1.21 and the NAb validation screening cut point of 0.587. Of the 140 screened samples, 34% (48 samples) screened negative in both the TAb and NAb assays (TAb-/NAb-), whereas 55% (77 samples) screened positive in both assays (TAb+/NAb+). Five samples (3.6%) that screened positive in the TAb assay did not demonstrate positivity for neutralizing antibodies to AAVrh.10 (TAb+/NAb-) (Table VII). Interestingly, 7% (10 samples) screened positive with varying responses in the NAb assay, although they screened negative in the TAb assay (TAb-/NAb+). Seven of the positive screened NAb samples were further assayed in the NAb titration assay to determine a sample titer to understand the level of NAb in these samples. The three other positive NAb sample volumes were exhausted and were not able to be tittered in the assay. The titer of these 7 samples ranged from dilution factors of <4, 4, 8, 16, 32, and 64 (Fig. 7).

**Table VII:** Concordance of TAb and NAb Data

		Anti-AAVrh.10 Neutralizing Antibodies (NAb)	
		Negative	Positive
Anti-AAVrh.10 Total Antibodies (TAb)	Positive	5	77
	Negative	48	10

The five samples that screened positive in the TAb assay and negative in the NAb assay (TAb+/NAb-) were further titered in the TAb assay (Supplemental Table S1). Four of the five samples that screened positive in the TAb assay tested negative when titrated. This was expected given the low positivity of the those screened samples (within 2-fold of the assay cut point). It is important to note that these samples could have been false positives as the screening cut point was determined with a 5% FPR whereas the titer cut point was determined at a 1% FPR when the samples were tittered. These reasons could explain why these samples initially screened positive for TAb and were negative for NAb. In addition, the higher sensitivity of the TAb assay could be a reason why the samples did not screen positive in the NAb assay, as demonstrated with sample 2 (Fig. 7). A Cohen’s Kappa test, a statistic that is commonly implemented in inter-rater reliability studies, was used to assess the extent of agreement between categorical results of the two assays and was calculated to be 0.78, representing substantial agreement in results between the assays.

## Discussion

Oftentimes when a biomarker, genotype, or other measurable feature is used as a decision point to include or exclude a patient for treatment in a clinical trial, the assay for its detection is also developed as a companion diagnostic test (CDx) to be used as a criterion for treatment once the therapeutic is approved. In the case of AAV gene therapies, using the anti-AAV antibody titer assays as an inclusion/exclusion criterion for enrollment suggests the assay potentially may need to be carried forward as a companion diagnostic, especially since there is a lack of data for treating patients who were excluded from clinical studies with those criteria, posing an efficacy and safety risk. Choosing which assay result, TAb or NAb, to use as the inclusion/exclusion criterion is therefore an important consideration.

Anti-AAV antibody titers can be measured in total antibody assays and in neutralizing antibody assays. TAbs measure all the binding antibodies, whereas NAb assays measure a subset of total antibodies that neutralize the activity of the capsid transduction and contribute to loss of efficacy in therapeutics. TAb assays are typically easier to deploy, mainly due to their robustness, high-throughput capability, and decreased variability compared to NAb assays. Generally, NAb assays are cell-based that are developed and validated based on the mechanism of action of the therapeutic. In the case of AAV-based gene therapies, the transduction of the AAV vector into cells for delivery of the transgene may be considered as the mechanism of action. AAV NAb assays are also referred to as transduction inhibition assays and can be developed with different reporter gene formats. Recommendations for developing and validating TAb and NAb assays against AAV have been extensively described [26, 27]. Current discussions in the industry have suggested that due to their robust nature, TAb assays could be useful as valuable alternatives to NAb assays for the detection of anti-AAV antibodies, especially in cases where a functional assay is difficult to develop, or the mechanism of action is not fully elucidated.

Previous studies have demonstrated the prevalence of total and neutralizing antibodies to common AAV serotypes in humans. In an earlier study, the authors showed from 100 healthy human samples, the prevalence of total antibodies to AAVrh.10 to be 59% with 21% of that number being neutralizing antibodies to AAVrh.10, demonstrating most anti-AAVrh.10 IgG were not neutralizing [28]. Similarly, in 140 screened human samples, our findings suggest 58.6% seroprevalence of total antibodies to AAVrh.10, but we show a 62.1% seroprevalence of neutralizing antibodies, suggesting that that most anti-AAVrh.10 IgG were neutralizing. The difference in serotype prevalence of AAVrh.10 neutralizing antibodies could be due to the different assay formats, sensitivity and cut point determinations between the neutralizing antibody assays. In addition, this study only included serum samples from male donors only due to supply chain challenges during the pandemic. Several studies have shown comparisons and impacts of using different TAb assay formats (colorimetric versus chemiluminescent ELISA) and neutralizing antibody assay formats (GFP-based versus luciferase-based) [29, 30, 31, 32]. Variations in assay format and sensitivity could impact subject enrollment, and thus it is important to compare assay formats and understand the reason for differences in sample responses between assays. In addition, to our knowledge, the only other study that compared anti-AAVrh.10 TAb and NAb antibodies was done for a non-human primate study assessing if a humoral response was evoked by either intraventricular or intracisternal central nervous system delivery of an AAVrh.10 gene therapy [33]. In this study, they demonstrated a comparison between either of the administration routes, intracisternal and intraventricular, and showed comparable post-treatment TAb and NAb titers regardless of the route of administration; whereas they observed no significant TAb or NAb titers in the non-treated NHPs post administration [33].

This publication is the first to our knowledge that compares methods for detection of TAbs and NAbs to AAVrh.10 in human serum. When comparing assay sensitivity for both assays, the TAb assay was more sensitive than the NAb assay with determined sensitivity values of 8.9 ng/mL and 77.7 ng/mL, respectively. However, the NAb assay demonstrated better sensitivity in comparison to most cell based NAb assays that generally struggle to meet sensitivity values of <100 ng/mL that are routinely reached with TAb assays [34]. In addition, our observation of 10 samples that were positive for NAb and negative for TAb responses suggests that the NAb assay is highly sensitive or susceptible to non-antibody inhibitory factors. This observation has also been seen with other AAV serotype NAb assays, suggesting that uncharacterized serum components can inhibit AAV transduction and corroborating the idea that NAb assays may be susceptible to other inhibitory factors that are not necessarily linked to antibody activity [30, 39]. We have shown comparability of our TAb and NAb assays when screening 140 human serum samples with relatively comparable seroprevalence for TAb and NAb antibodies to AAVrh.10, 58.6% and 62.1% respectively. Thus, suggesting if we used either assay for inclusion/exclusion into a study, we do not risk screening out a larger number of patients by using one assay over the other.

With respect to clinical relevance, a recent study showed no impact of moderately higher NAb titers (pre-existing titers up to 340) on the transduction efficiency or efficacy of an intravenous delivery of an anti-AAV5 human factor IX gene therapy in a phase IIb trial for treatment of Hemophilia B [30]. In the same publication the data was corroborated in an NHP study that also demonstrated transduction efficiency for AAV5-hFIX in NHPs with pre-existing titers >1000 [30]. A summary of cutoff values that have been used in clinical studies using different AAV serotypes and transgenes in a variety of diseases as described in a recent publication [35] shows a range of cutoff values from 1:5 to 1:400 using different assay formats (TAb versus NAb). The lack of data for using less conservative cutoff values >1:50 begs the question whether we truly understand AAV gene therapy effectiveness for individuals with moderately higher titers. Furthermore, there is also a lack of understanding of not only the effect of pre-existing anti-AAV antibodies but also the effect of an immune response post administration to AAV gene therapies on safety and efficacy and implications on repeated dosing with these therapies. This underscores the importance of properly characterizing and validating immunogenicity assays for monitoring immune response for AAV-based gene therapy clinical studies. Our results suggest a strong agreement between both the validated TAb and NAb assays, with either assay being suitable for use for patient enrollment in AAVrh.10 gene therapy clinical studies. If either assay were to be used for patient inclusion/exclusion and eventually developed as a CDx for an AAVrh.10 gene therapy in the Unites States, the assay would need to be under regulation by the Center for Devices and Radiological Health (CDRH) and performed in a Clinical Laboratory Improvement Amendments (CLIA)-compliant laboratory [19]. An optimal CDx assay would need to be robust and maintain stable performance over several years which lends an advantage of using a TAb assay as a CDx. In addition, TAb assays are more sensitive and more specific than NAb or transduction inhibition (TI) assays. As demonstrated in this manuscript and others, the source of positivity of NAb samples from TI assays could be from antibody or non-antibody sources and would need to be confirmed as a neutralizing antibody by depletion with immunoglobulin. This non-specificity of NAb assays adds another layer of complexity if carried forward as a CDx. Using a TAb assay as a CDx is more conservative given you would detect all anti-AAV antibodies and thus limit the risk of enrolling patients who may not benefit from the gene therapy; however, this strategy could also potentially screen out and exclude patients who could benefit from the treatment.

## Conclusion

With the rise in AAV gene therapies, we are seeing an increase in data that allows us to understand the levels of pre-existing anti-AAV antibodies in the population, and clinical trial screening for pre-existing antibodies allows us to understand efficacy in low titer patients. This data is more available for the common serotypes like AAV2, AAV5 and AAV9 and less so for AAVrh.10. With the lack of information on AAVrh.10 it is important to develop and validate assays for detection of total and neutralizing anti-AAVrh10 antibodies to understand the concordance between both formats and the impact of pre-existing AAVrh.10 antibodies on efficacy and safety of AAVrh.10 gene therapies. As gene therapy trials continue to rise, along with the potential use of the AAVrh.10 serotype in gene therapy products, the information we present herein suggests a concordance between TAb and NAb assays that demonstrates the suitability of either to be used as a screening assay for patient enrollment into AAVrh.10 gene therapy clinical studies. This information contributes to the acceleration of drug development and provides the industry with information on assays that can be used for patient enrollment and as a potential companion diagnostic for AAVrh.10 gene therapies.

### Supplementary Information

ESM 1 (DOCX 176 kb)
