# Supplementary material for: Validation of Anti-Adeno Associated Virus Serotype rh10 (AAVrh.10) Total and Neutralizing Antibody Immunogenicity Assays
Source: Pharm Res. 2023 Oct 25;40(10):2383–97. doi: 10.1007/s11095-023-03625-7 (PMC10661749; doi:10.1007/s11095-023-03625-7)
Supplement: Supplementary file 1 — (DOCX 176 kb) [file 11095_2023_3625_MOESM1_ESM.docx]

**Supplemental Table S1: Titration of Positive TAb/Negative NAb Samples in the anti-AAVrh.10 Total Antibody Assay**

|  | | **S1** | | **S2** | | **S3** | | **S4** | | **S5** | |
| --- | --- | --- | --- | --- | --- | --- | --- | --- | --- | --- | --- |
| **Dilution in Normal Human Serum** | **Dilution at MRD 10** | **S/N** | **CV (%)** | **S/N** | **CV (%)** | **S/N** | **CV (%)** | **S/N** | **CV (%)** | **S/N** | **CV (%)** |
| Neat | 10 | 1.163 | 7.7 | 2.083 | 3.1 | 1.047 | 2.4 | 1.015 | 3.9 | 1.191 | 2.5 |
| 2 | 20 | 0.983 | 9.6 | 1.487 | 3.4 | 0.969 | 3.6 | 1.012 | 0.5 | 1.033 | 5.3 |
| 4 | 40 | 0.959 | 4.2 | 1.163 | 0.9 | 0.952 | 1.0 | 1.004 | 2.5 | 0.994 | 1.0 |
| 8 | 80 | 0.916 | 3.3 | 1.078 | 1.8 | 0.955 | 0.5 | 0.962 | 6.7 | 1.022 | 2.9 |
| 16 | 160 | 0.902 | 0.0 | 1.015 | 2.0 | 0.934 | 0.5 | 1.015 | 2.9 | 0.983 | 2.5 |
| 32 | 320 | 0.923 | 1.1 | 1.026 | 0.5 | 0.899 | 2.8 | 0.969 | 1.5 | 1.026 | 0.5 |
| 64 | 640 | 0.962 | 0.5 | 1.001 | 3.0 | 0.973 | 3.1 | 1.110 | 12.1 | 1.001 | 0.0 |
| 128 | 1280 | 0.962 | 3.6 | 1.012 | 3.4 | 1.004 | 0.5 | 1.015 | 4.9 | 1.036 | 2.9 |
| Bold Shaded indicates ≥ TCP (1.40) | | | | | | | | | | | |

Five normal serum samples (denoted S1-S5) that screened positive in the anti-AAVrh.10 TAb assay and negative in the NAb assay were tittered in the TAb assay. Each sample was diluted in normal human serum pool (NC) in a two-fold dilution series, then an MRD of 1:10 was applied to each sample. The mean normalized signal and %CV for each sample dilution is displayed in the table.
